# Supplementary material for: Targeting mutant RAS in patient-derived colorectal cancer organoids by combinatorial drug screening
Source: eLife. 2016 Nov 15;5:e18489. doi: 10.7554/eLife.18489 (PMC5127645; doi:10.7554/eLife.18489)
Supplement: Figure 4—source data 1. — A number of biological replicates for each dose-response curve are indicated between parenthesis (first monotherapy/ second monotherapy/ combination therapy). DOI: http://dx.doi.org/10.7554/eLife.18489.016 [file elife-18489-fig4-data1.pdf]

Figure 4-Source data 1

# Combination therapy: EGFRi & MEKi

P18T | P18T $KRAS^{G12D}$

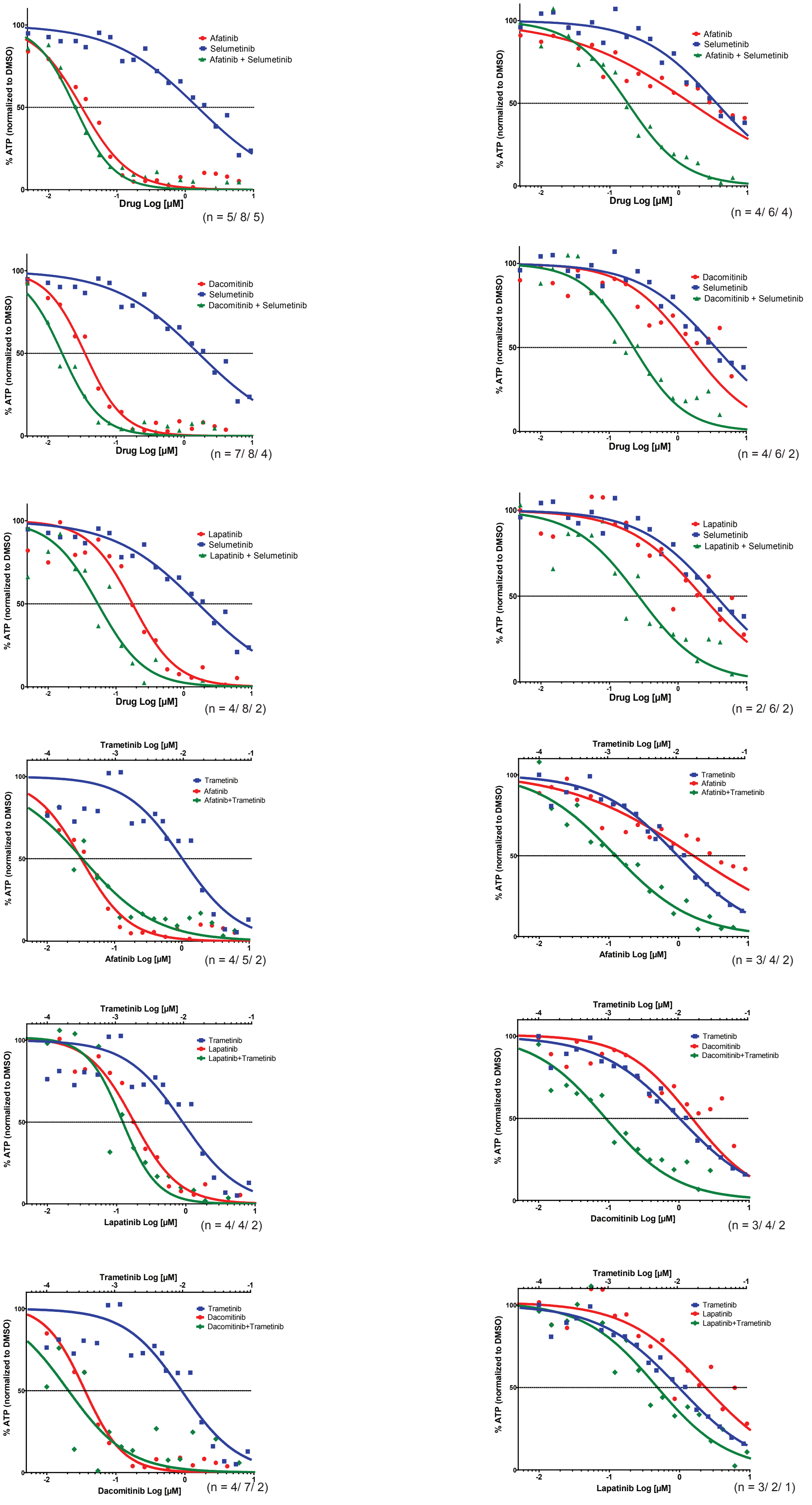

# Combination therapy: MEKi & ERKi

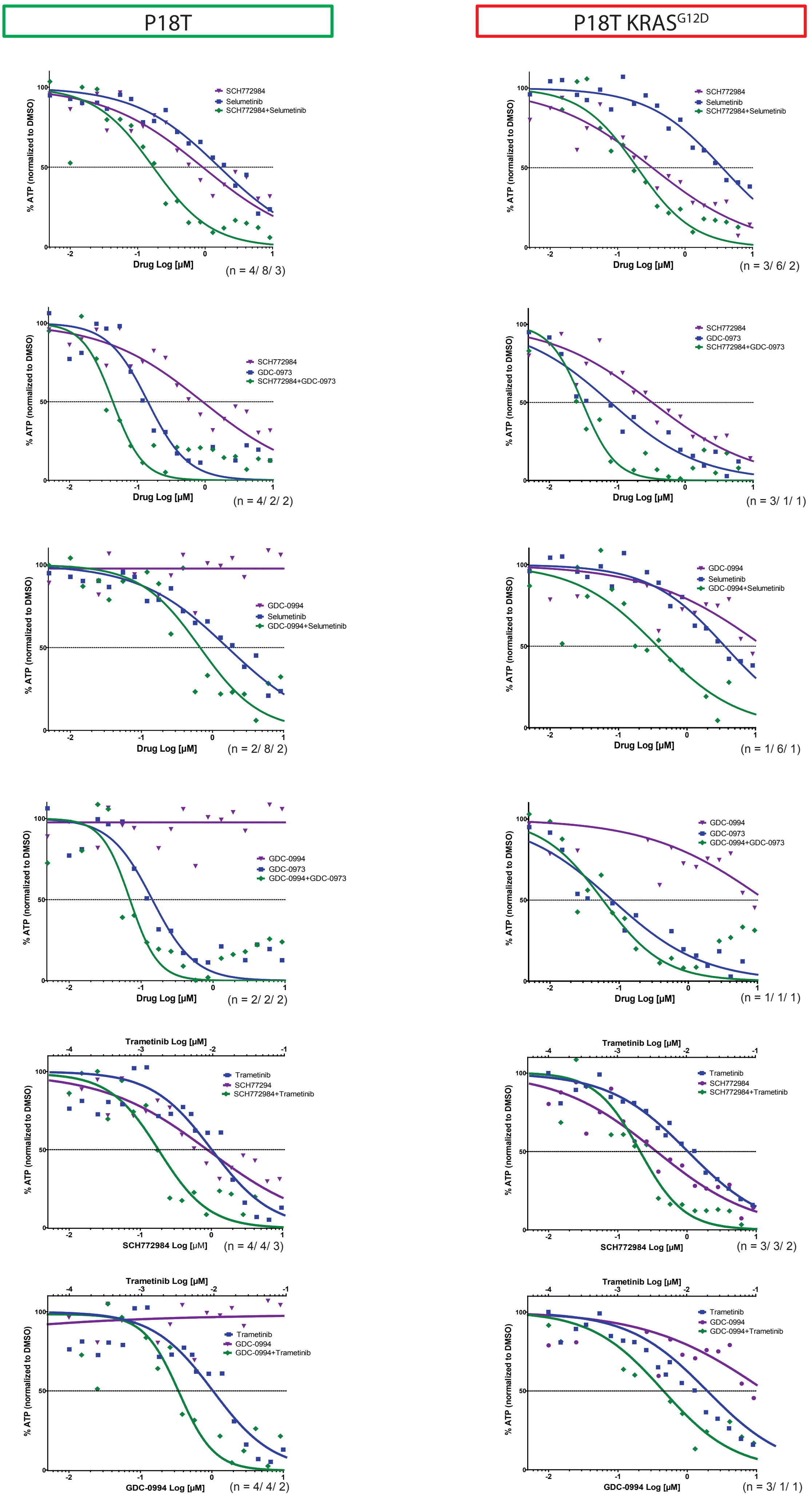

# Combination therapy: EGFRi & ERKi

| P18T | P18T KRAS$^{G12D}$ |
| --- | --- |

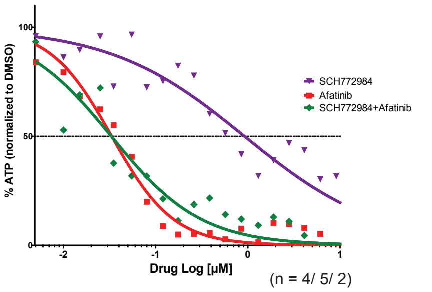


(n = 4/ 5/ 2)

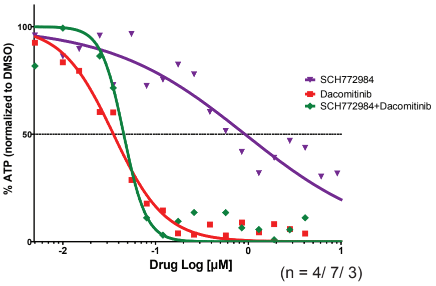


(n = 4/ 7/ 3)

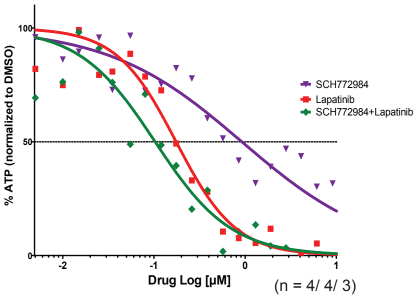


(n = 4/ 4/ 3)

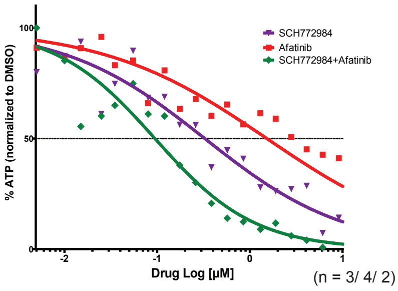


(n = 3/ 4/ 2)

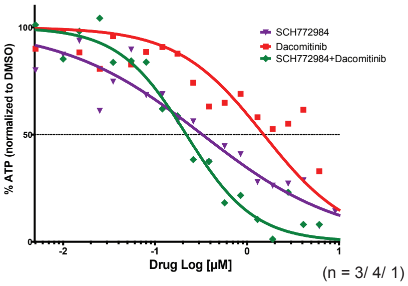


(n = 3/ 4/ 1)

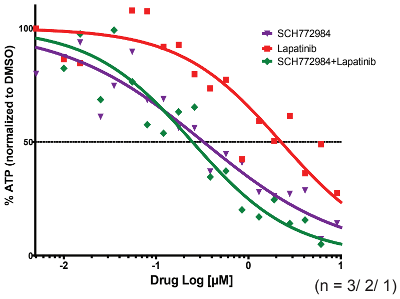


(n = 3/ 2/ 1)
