# Supplementary material for: Targeting mutant RAS in patient-derived colorectal cancer organoids by combinatorial drug screening
Source: eLife. 2016 Nov 15;5:e18489. doi: 10.7554/eLife.18489 (PMC5127645; doi:10.7554/eLife.18489)

Figure 4-Source data 2

Combination therapy: EGFRi & AKTi

P18T | P18T KRAS^{G12D}

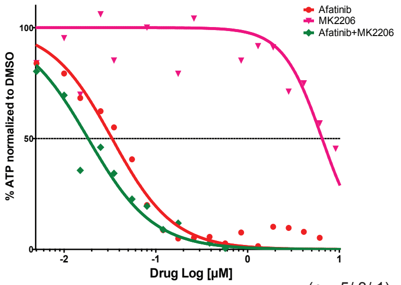

(n = 5/ 3/ 1)

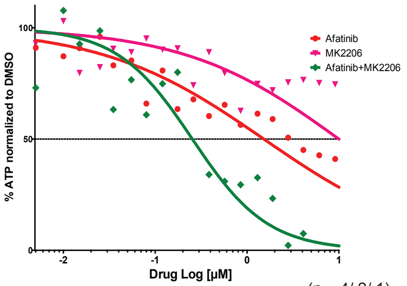

(n = 4/ 2/ 1)

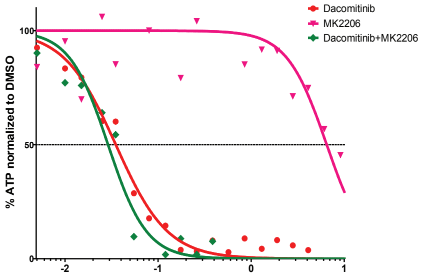

(n = 7/ 3/ 2)

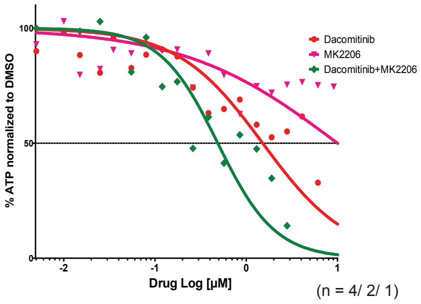

(n = 4/ 2/ 1)

Combination therapy: EGFRi & PI3Ki

P18T | P18T KRAS^{G12D}

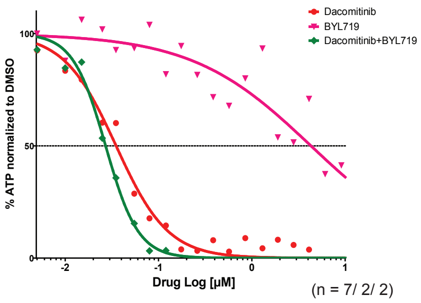

(n = 7/ 2/ 2)

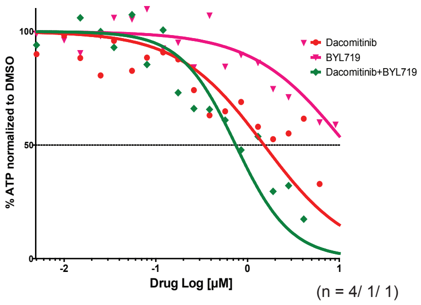

(n = 4/ 1/ 1)

Combination therapy: EGFRi & MEKi & AKTi

P18T | P18T KRAS^{G12D}

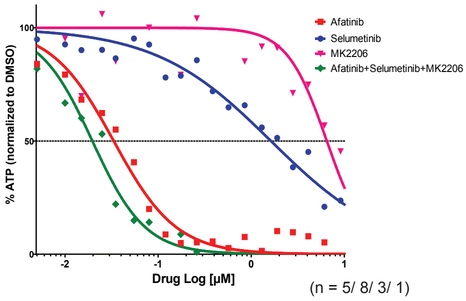

(n = 5/ 8/ 3/ 1)

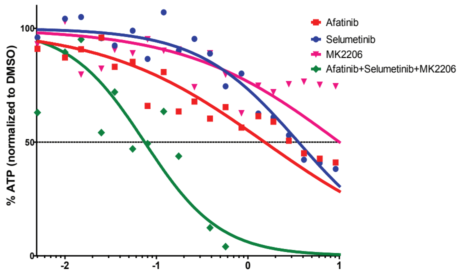

(n = 4/ 6/ 2/ 1)

Combination therapy: MEKi & AKTi

P18T

P18T KRAS^{G12D}

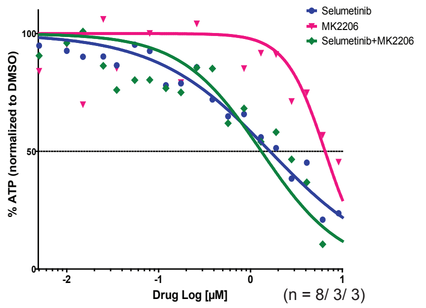

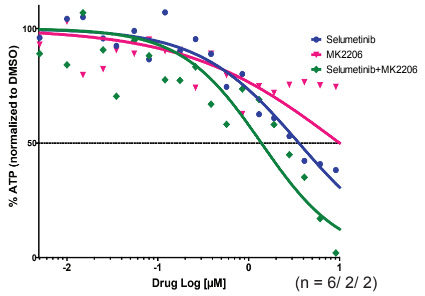

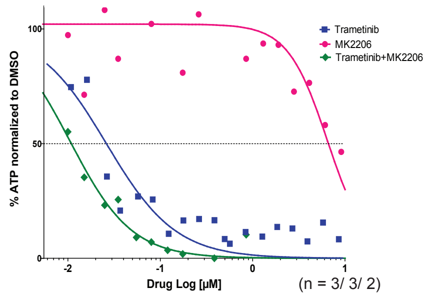

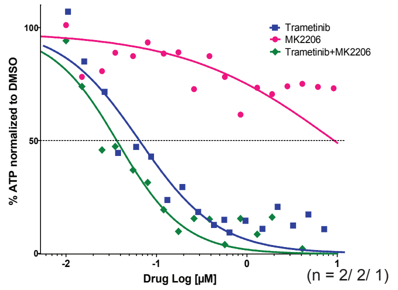

Combination therapy: MEKi & PI3Ki

P18T

P18T KRAS^{G12D}

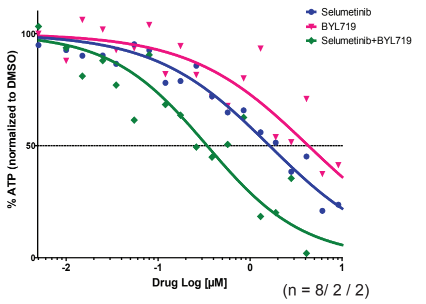

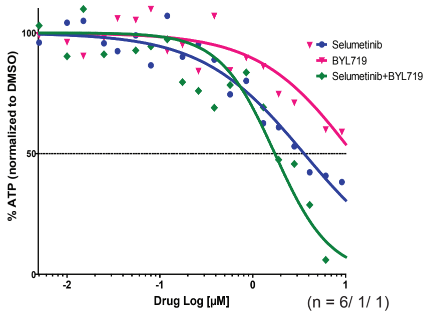

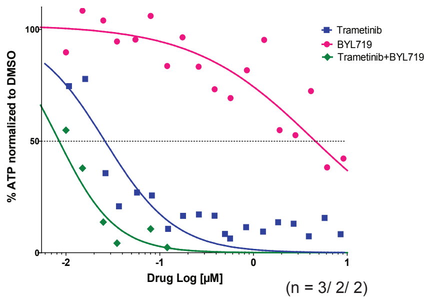

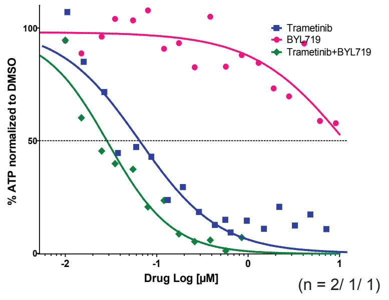

Supplement: Figure 4—source data 2. — A number of biological replicates for each dose-response curve are indicated between parenthesis (first monotherapy/ second monotherapy/ combination therapy). DOI: http://dx.doi.org/10.7554/eLife.18489.017 [file elife-18489-fig4-data2.pdf]
