# Supplementary material for: Targeting mutant RAS in patient-derived colorectal cancer organoids by combinatorial drug screening
Source: eLife. 2016 Nov 15;5:e18489. doi: 10.7554/eLife.18489 (PMC5127645; doi:10.7554/eLife.18489)

Figure 5-Source data 3

Combination therapy: EGFRi & MEKi

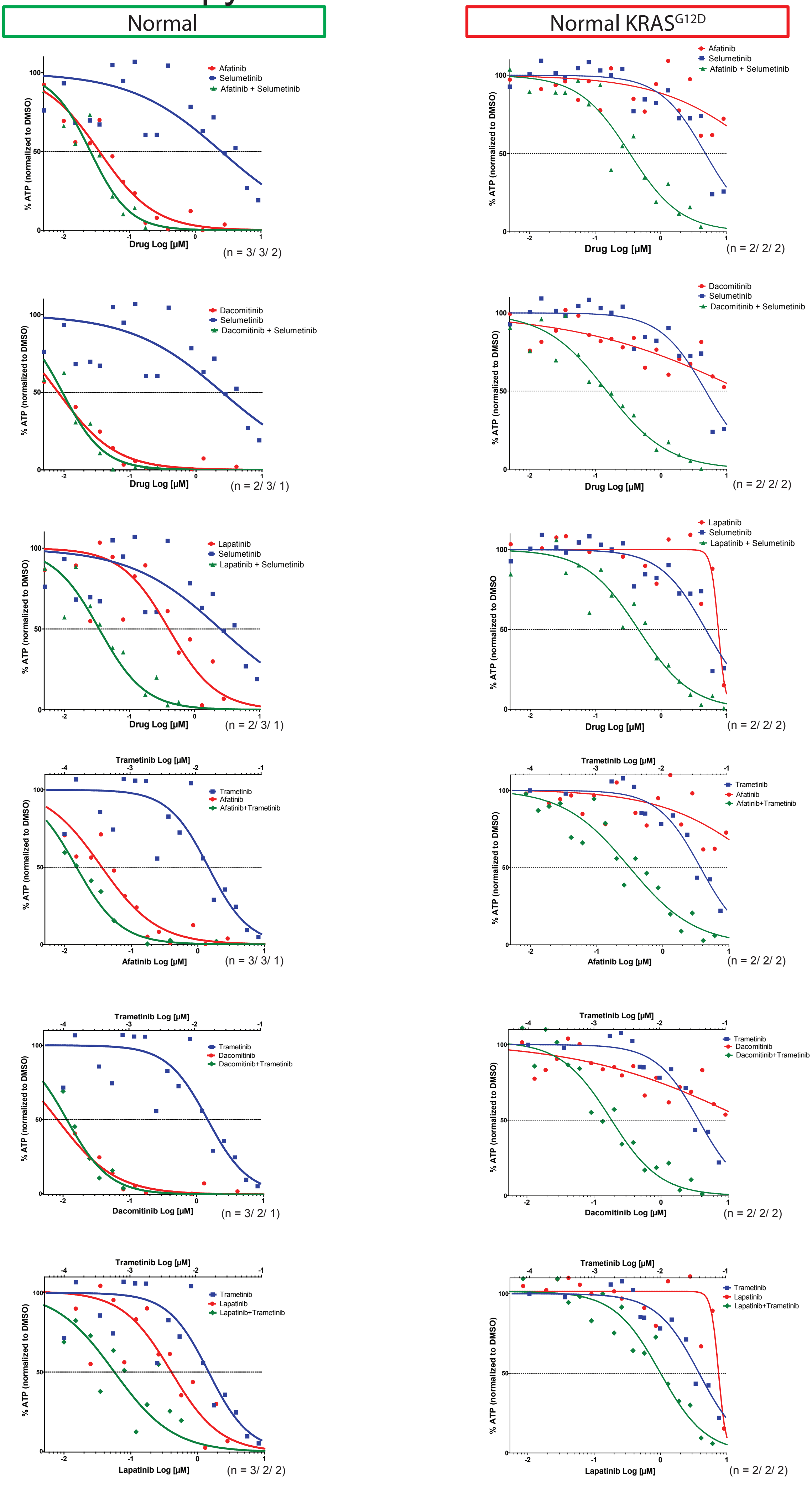

Combination therapy: MEKi & ERKi

Normal | Normal KRAS^{G12D}

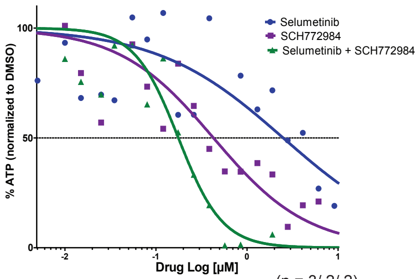

(n = 3/ 2/ 2)

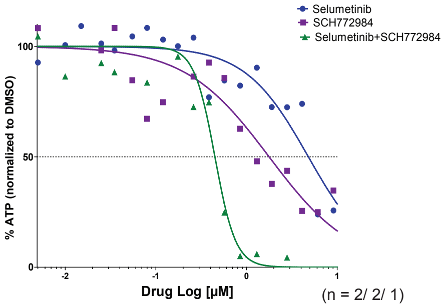

(n = 2/ 2/ 1)

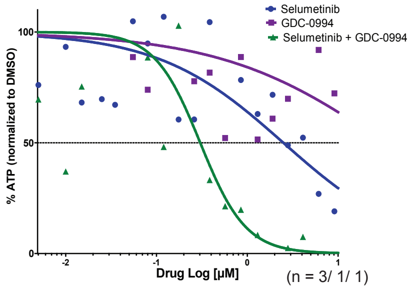

(n = 3/ 1/ 1)

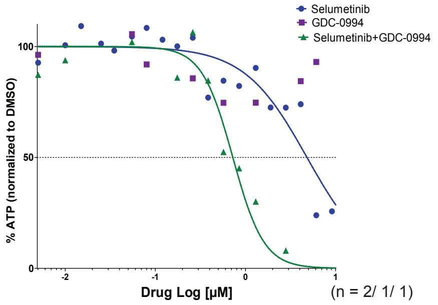

(n = 2/ 1/ 1)

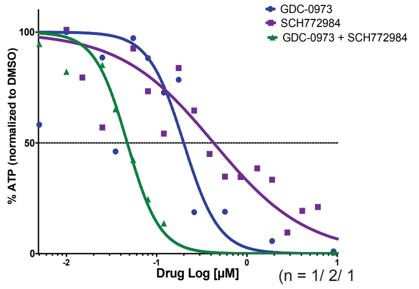

(n = 1/ 2/ 1

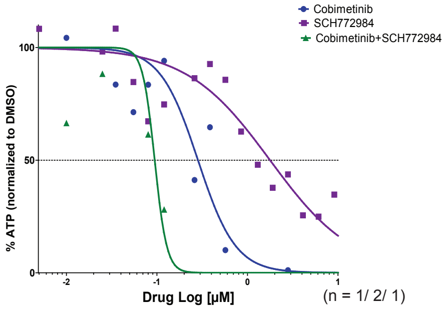

(n = 1/ 2/ 1)

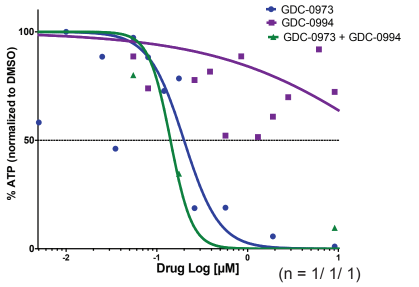

(n = 1/ 1/ 1)

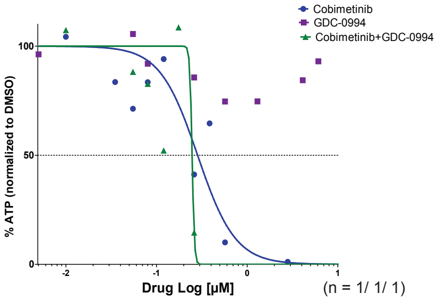

(n = 1/ 1/ 1)

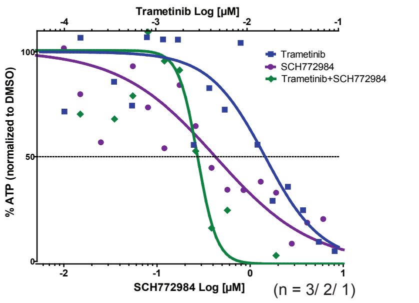

(n = 3/ 2/ 1)

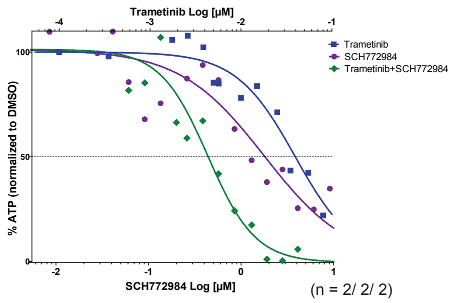

(n = 2/ 2/ 2)

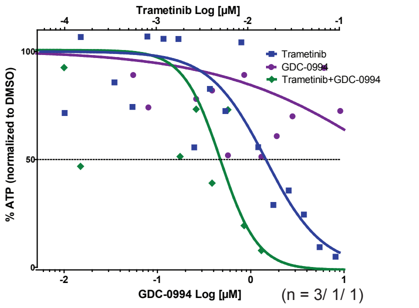

(n = 3/ 1/ 1)

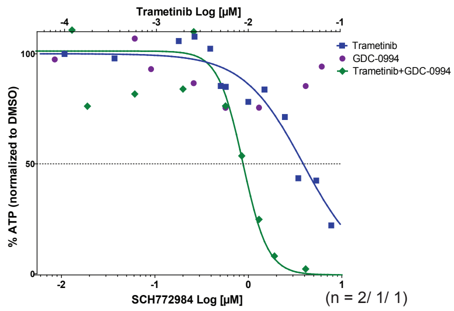

(n = 2/ 1/ 1)

Combination therapy: EGFRi & ERKi

Normal

Normal KRAS^{G12D}

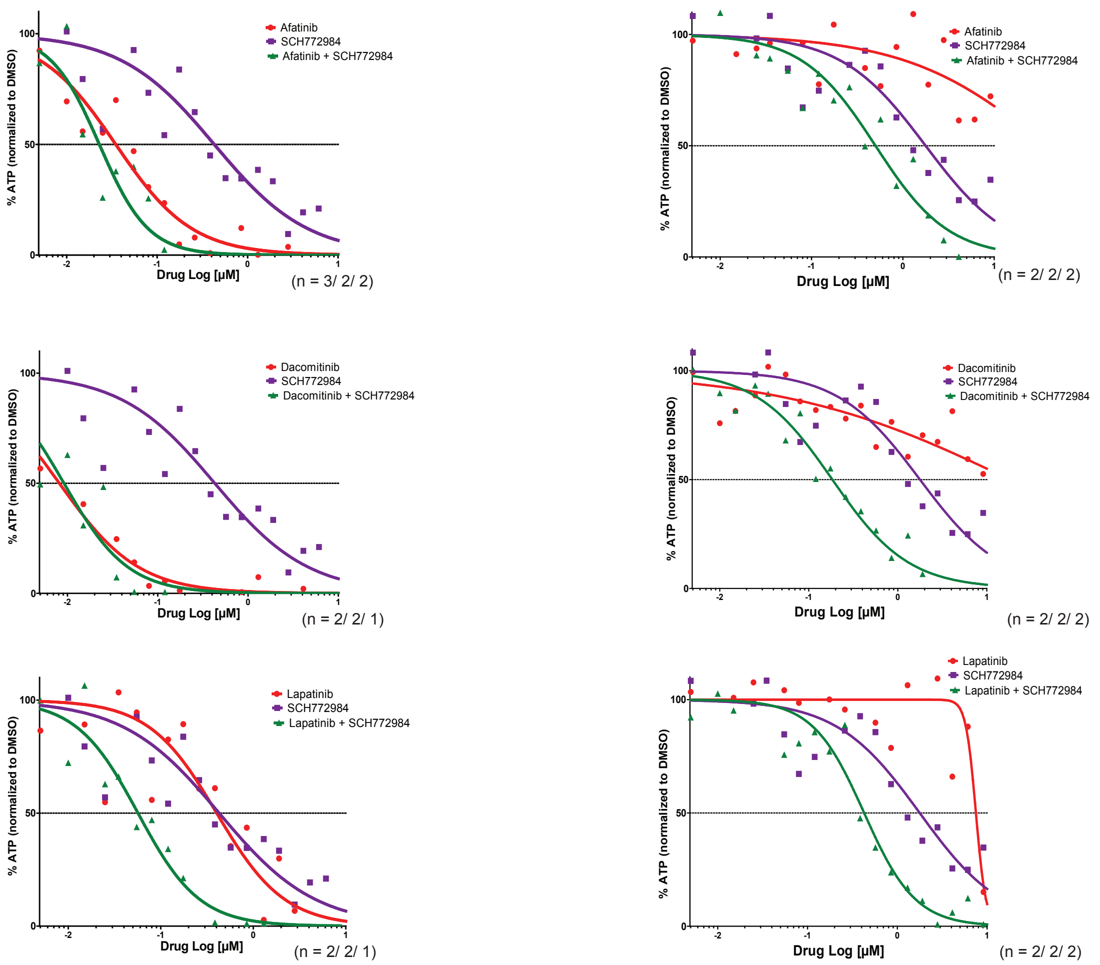

Supplement: Figure 5—source data 1. — Number of biological replicates for each dose-response curve are indicated between parenthesis (first monotherapy/ second monotherapy/ combination therapy). DOI: http://dx.doi.org/10.7554/eLife.18489.021 [file elife-18489-fig5-data1.pdf]
