# Supplementary material for: Targeting mutant RAS in patient-derived colorectal cancer organoids by combinatorial drug screening
Source: eLife. 2016 Nov 15;5:e18489. doi: 10.7554/eLife.18489 (PMC5127645; doi:10.7554/eLife.18489)

Figure 6-Source data 4

Combination therapy: EGFRi & MEKi

afatinib & selumetinib

KRAS mutant

P6T

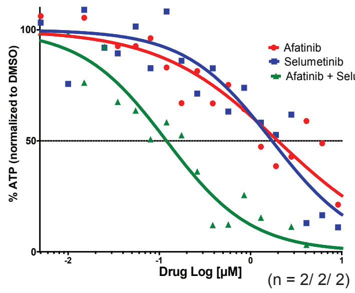

(n = 2/ 2/ 2)

P9T

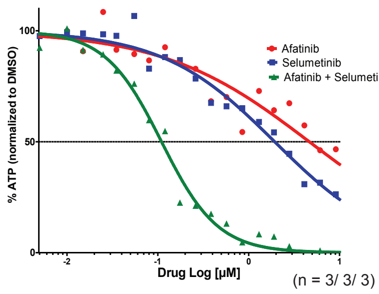

(n = 3/ 3/ 3)

P26T

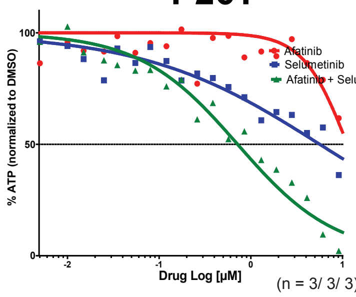

(n = 3/ 3/ 3)

P31T

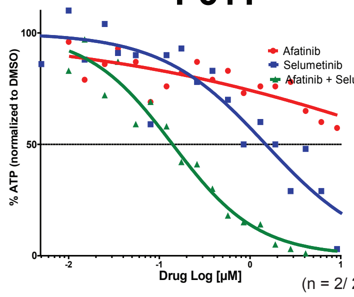

(n = 2/ 2/ 1)

BRAF mutant

P19bT

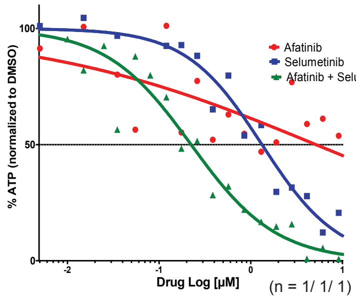

(n = 1/ 1/ 1)

NRAS mutant

P25T

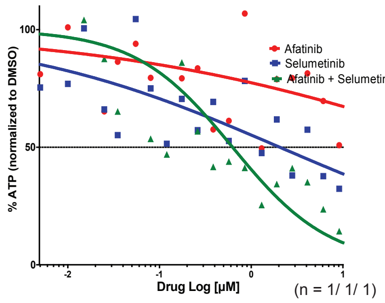

(n = 1/ 1/ 1)

KRAS WT

P8T

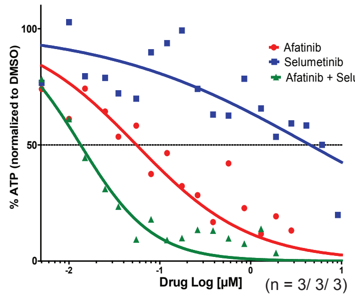

(n = 3/ 3/ 3)

P14T

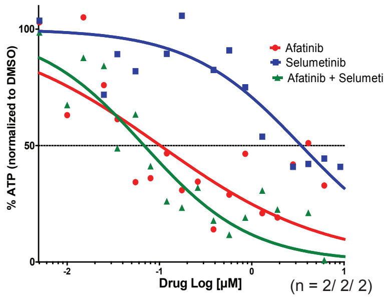

(n = 2/ 2/ 2)

P17T

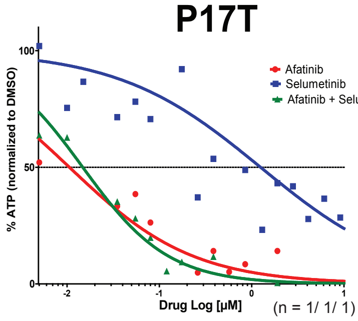

(n = 1/ 1/ 1)

P20T

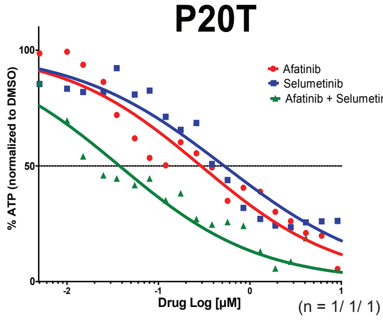

(n = 1/ 1/ 1)

Combination therapy: EGFRi & MEKi

dacomitinib & selumetinib

KRAS^{mutant}

P6T

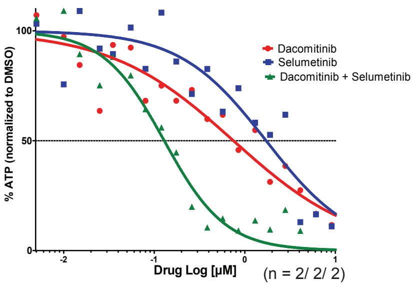

P9T

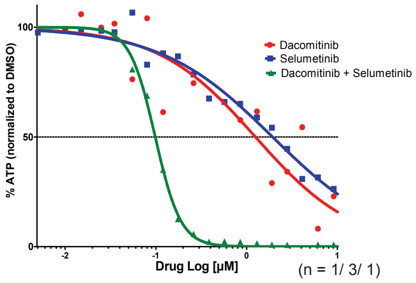

P26T

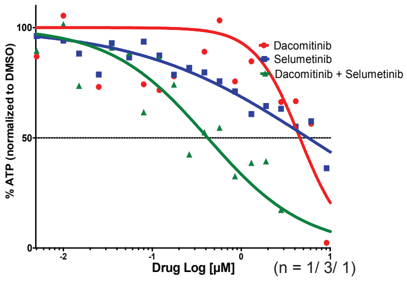

P31T

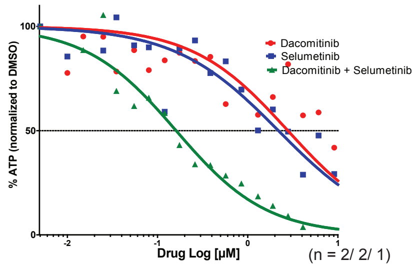

BRAF^{mutant}

P19bT

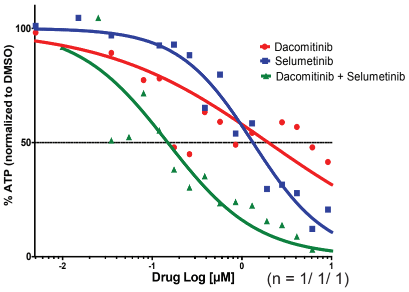

NRAS^{mutant}

P25T

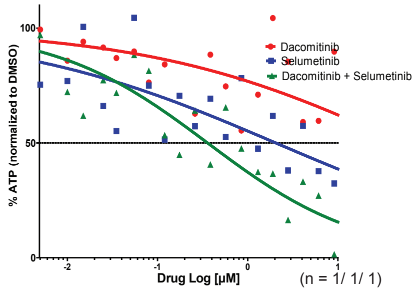

KRAS^{WT}

P8T

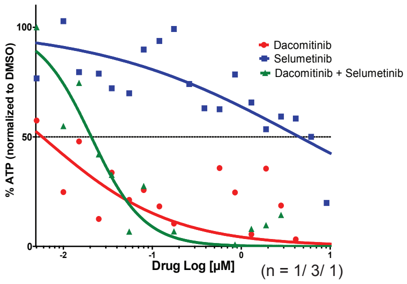

P14T

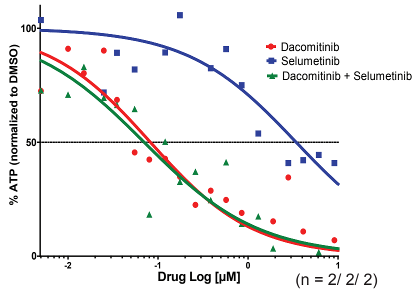

P17T

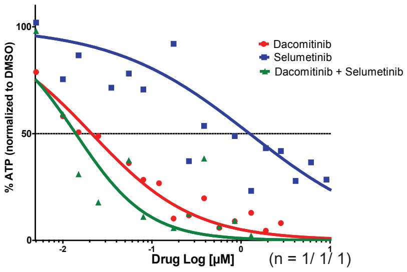

P20T

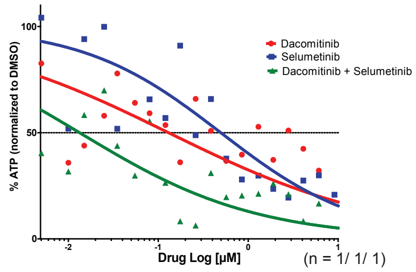

Combination therapy: EGFRi & MEKi

lapatinib & selumetinib

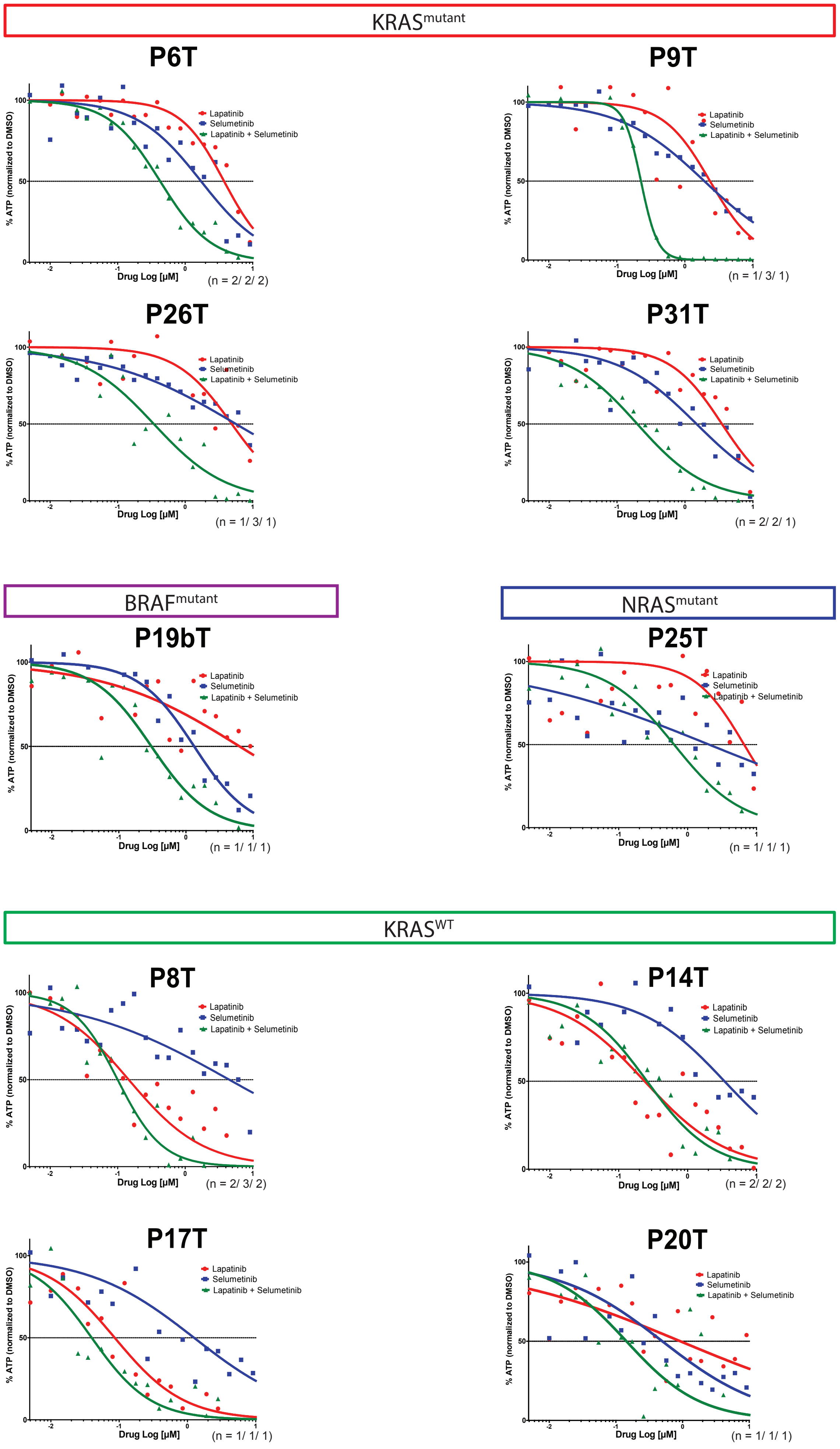

Combination therapy: MEKi & ERKi

selumetinib & SCH772984

KRAS^{mutant}

P6T

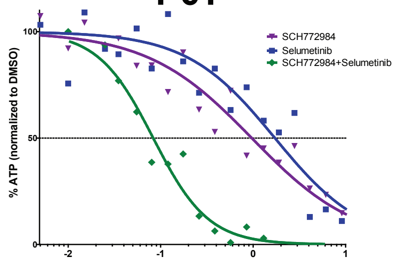

(n = 2/ 2/ 2)

P26T

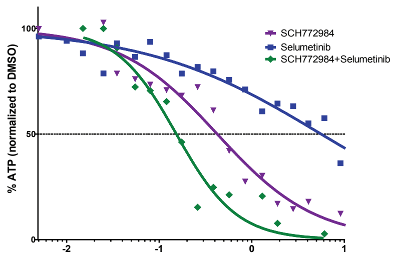

(n = 1/ 3/ 1)

P31T

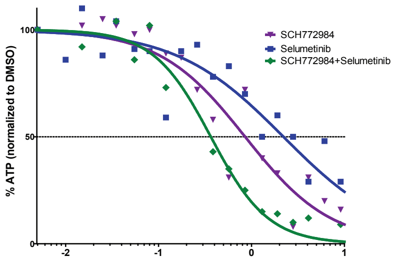

(n = 2/ 2/ 1)

BRAF^{mutant}

P19bT

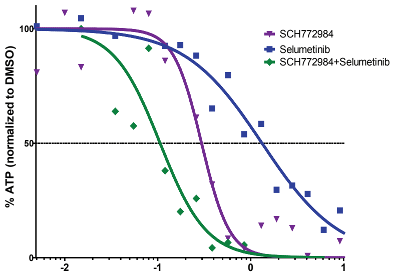

(n = 1/ 1/ 1)

NRAS^{mutant}

P25T

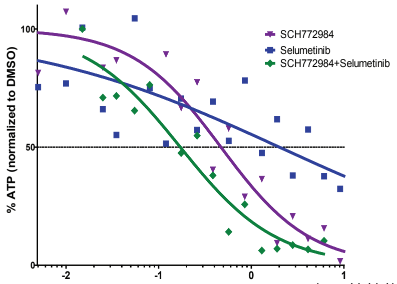

(n = 1/ 1/ 1)

KRAS^{WT}

P8T

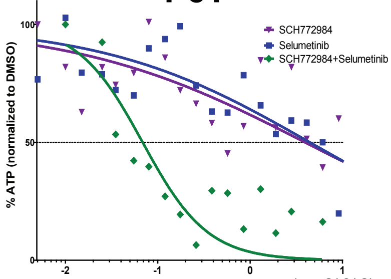

(n = 2/ 3/ 2)

P14T

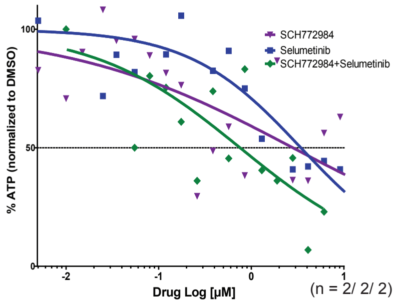

(n = 2/ 2/ 2)

P17T

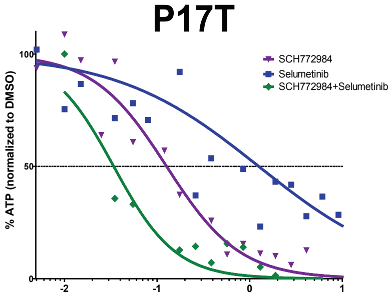

(n = 1/ 1/ 1)

P20T

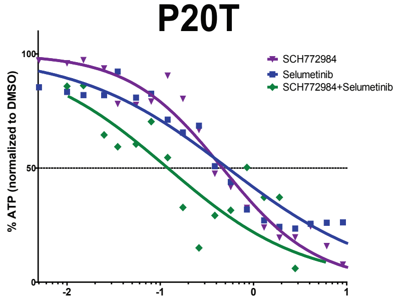

(n = 1/ 1/ 1)

Combination therapy: MEKi & ERKi

trametinib & SCH772984

KRAS^mutant

P6T

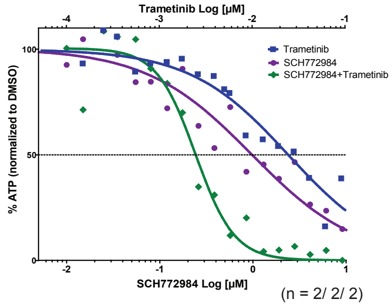

(n = 2/ 2/ 2)

P9T

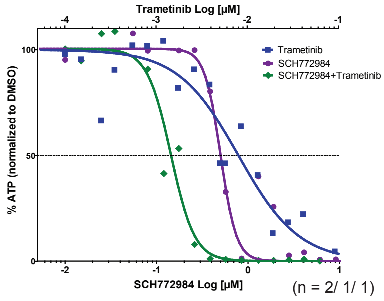

(n = 2/ 1/ 1)

P26T

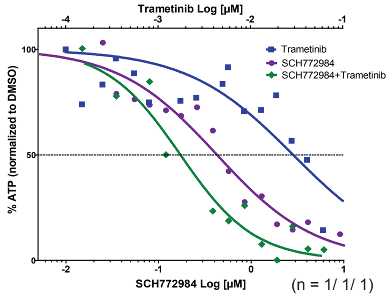

(n = 1/ 1/ 1)

P31T

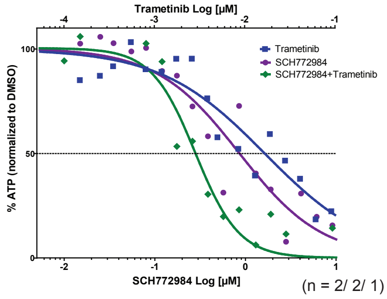

(n = 2/ 2/ 1)

BRAF^mutant

P19bT

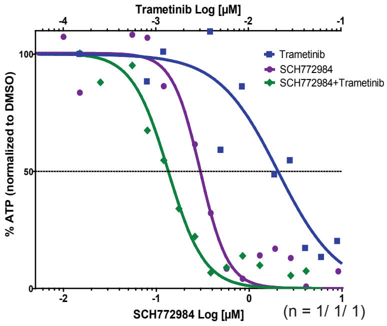

(n = 1/ 1/ 1)

NRAS^mutant

P25T

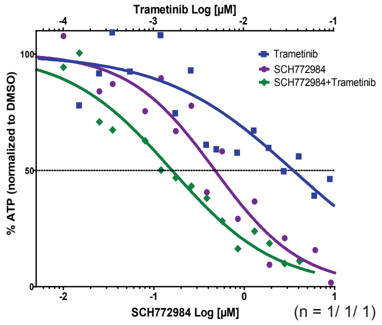

(n = 1/ 1/ 1)

KRAS^WT

P8T

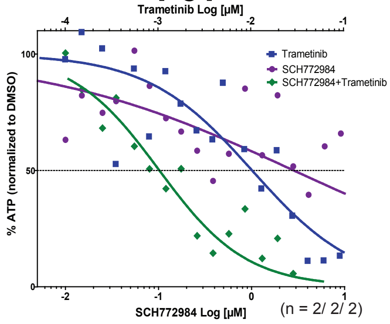

(n = 2/ 2/ 2)

P14T

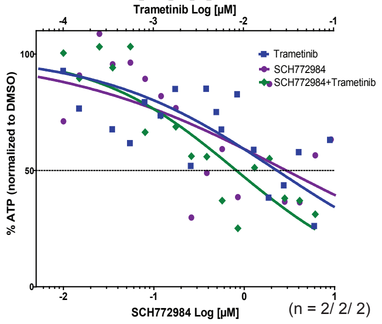

(n = 2/ 2/ 2)

P17T

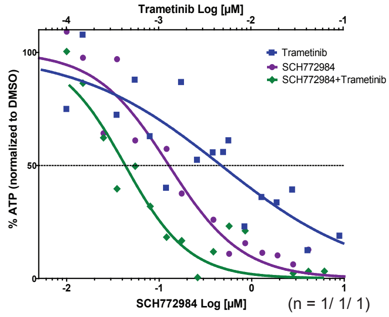

(n = 1/ 1/ 1)

P20T

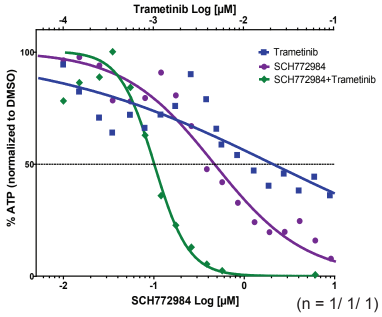

(n = 1/ 1/ 1)

Combination therapy: EGFRi & ERKi

afatinib & SCH772984

KRAS^{mutant}

P6T

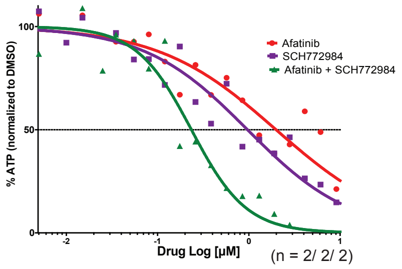

P9T

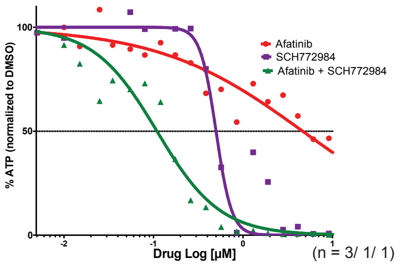

P26T

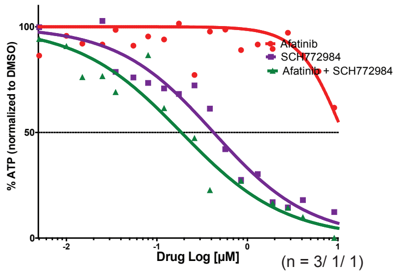

P31T

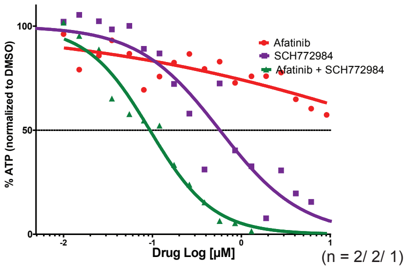

BRAF^{mutant}

P19bT

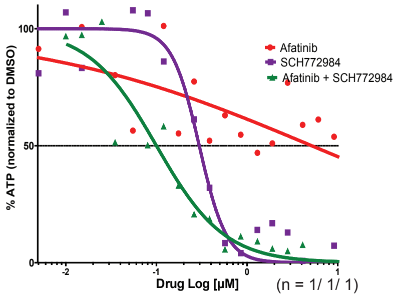

NRAS^{mutant}

P25T

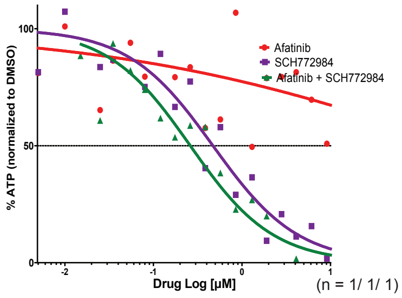

KRAS^{WT}

P8T

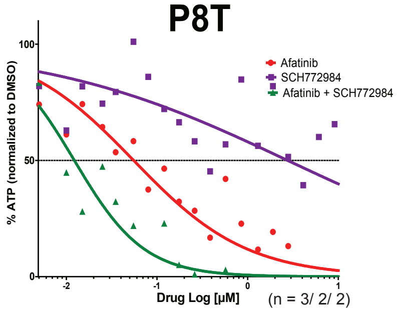

P14T

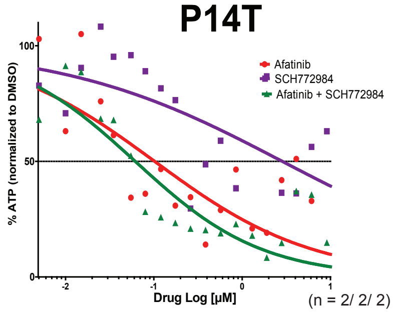

P17T

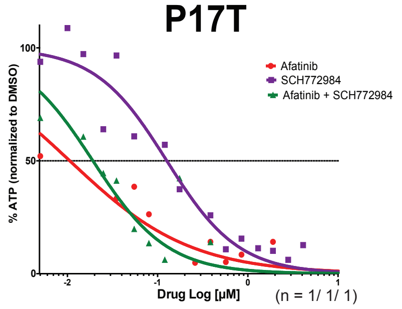

P20T

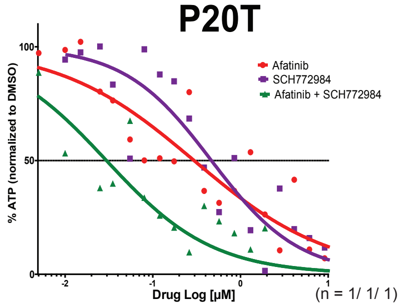

Combination therapy: EGFRi & ERKi

dacomitinib & SCH772984

KRAS^{mutant}

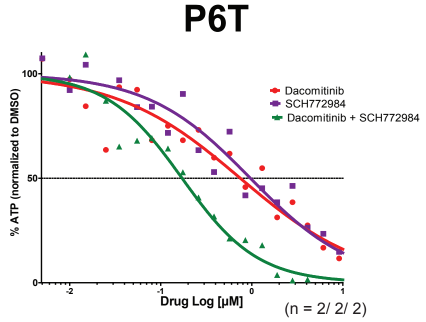

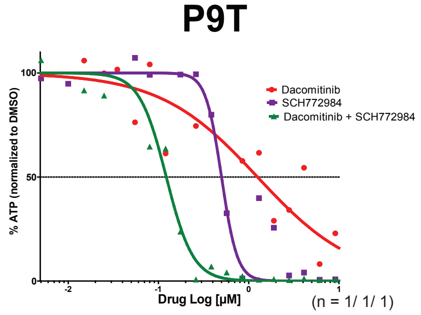

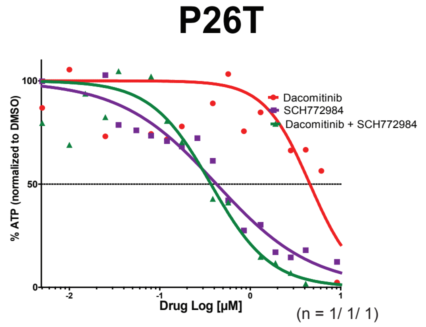

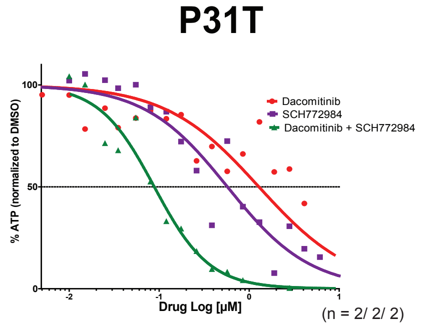

BRAF^{mutant}

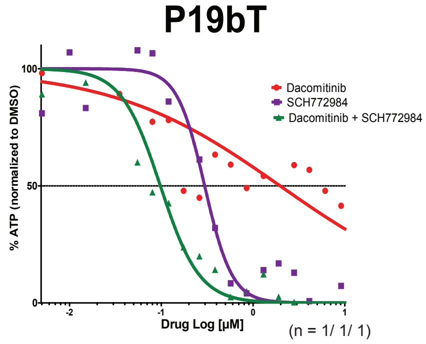

NRAS^{mutant}

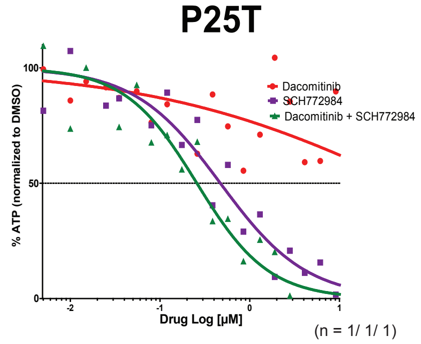

KRAS^{WT}

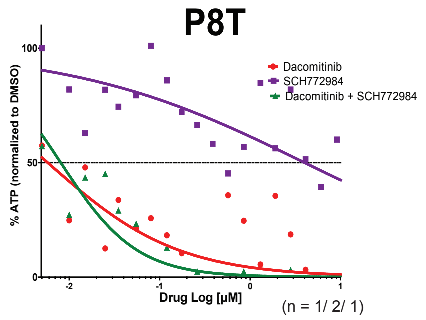

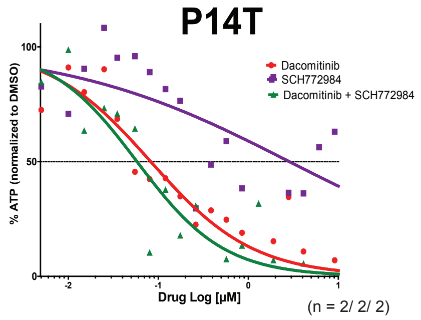

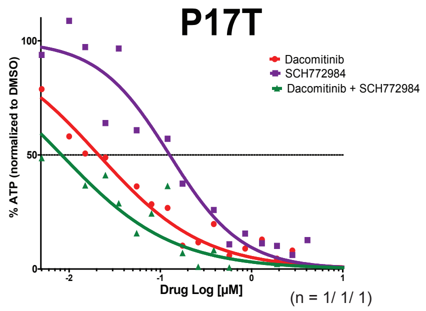

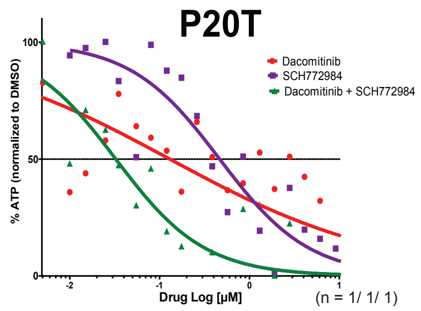

Combination therapy: EGFRi & ERKi

lapatinib & SCH772984

KRAS^{mutant}

P6T

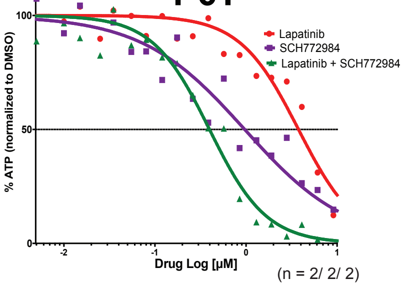

(n = 2/ 2/ 2)

P9T

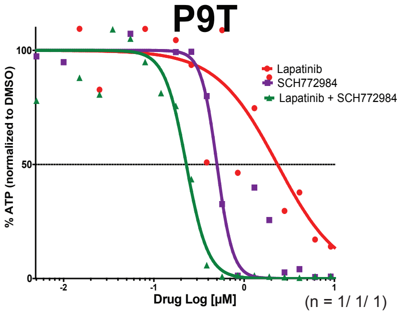

(n = 1/ 1/ 1)

P26T

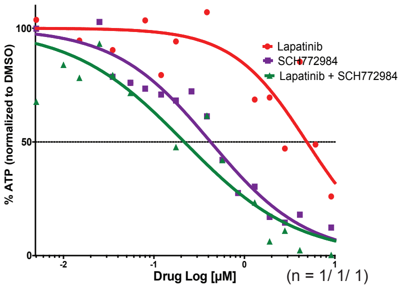

(n = 1/ 1/ 1)

P31T

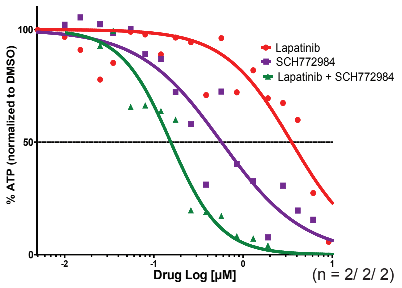

(n = 2/ 2/ 2)

BRAF^{mutant}

P19bT

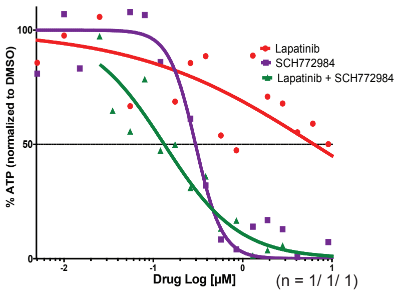

(n = 1/ 1/ 1)

NRAS^{mutant}

P25T

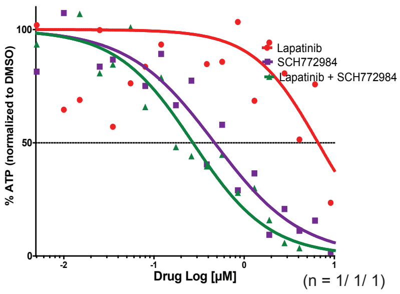

(n = 1/ 1/ 1)

KRAS^{WT}

P8T

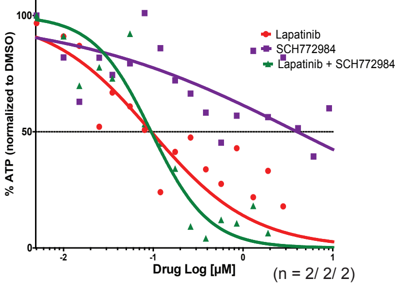

(n = 2/ 2/ 2)

P14T

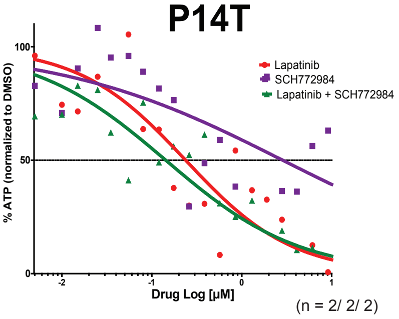

(n = 2/ 2/ 2)

P17T

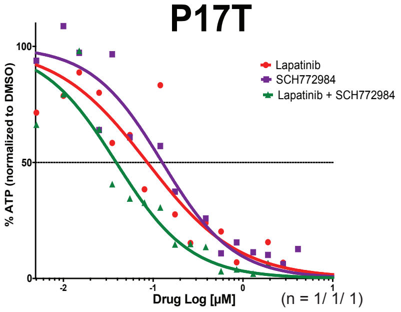

(n = 1/ 1/ 1)

P20T

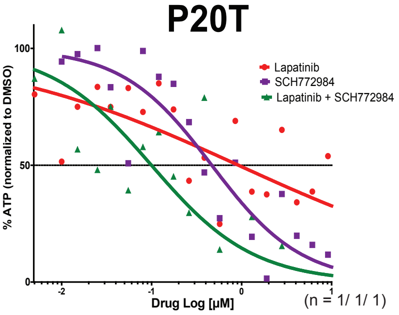

(n = 1/ 1/ 1)

Combination therapy: EGFRi & MEKi

afatinib & trametinib

KRAS^{mutant}

P6T

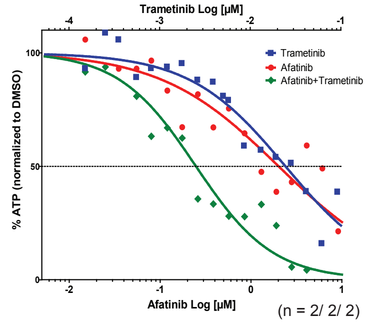

(n = 2/ 2/ 2)

P9T

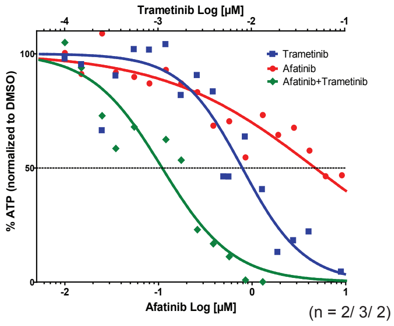

(n = 2/ 3/ 2)

P26T

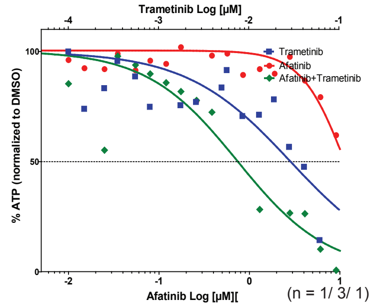

(n = 1/ 3/ 1)

P31T

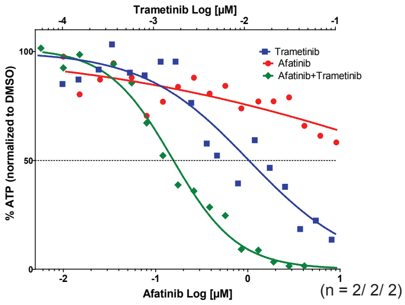

(n = 2/ 2/ 2)

BRAF^{mutant}

P19bT

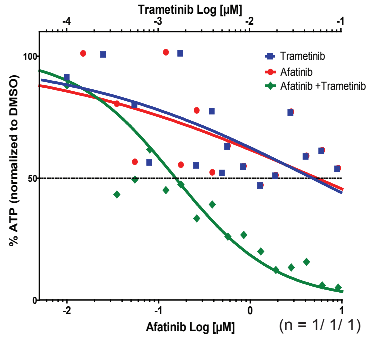

(n = 1/ 1/ 1)

NRAS^{mutant}

P25T

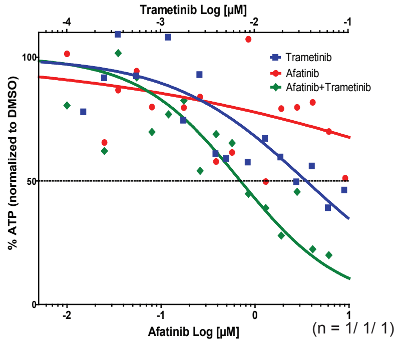

(n = 1/ 1/ 1)

KRAS^{WT}

P8T

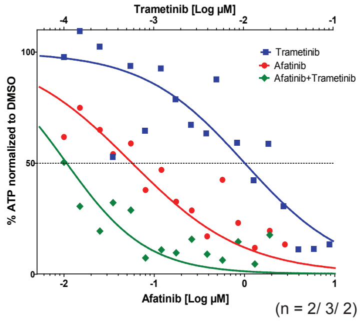

(n = 2/ 3/ 2)

P14T

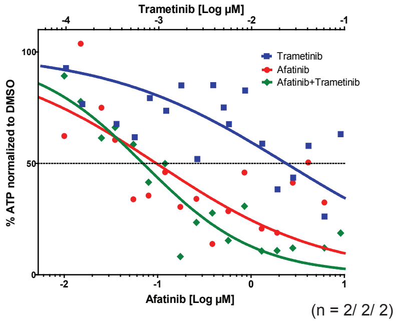

(n = 2/ 2/ 2)

P17T

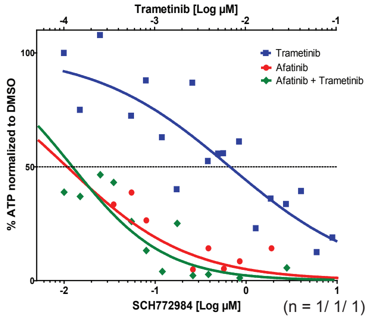

(n = 1/ 1/ 1)

P20T

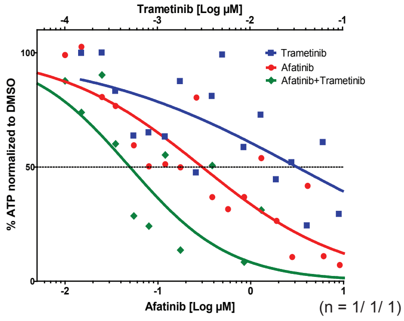

(n = 1/ 1/ 1)

Combination therapy: EGFRi & MEKi

dacomitinib & trametinib

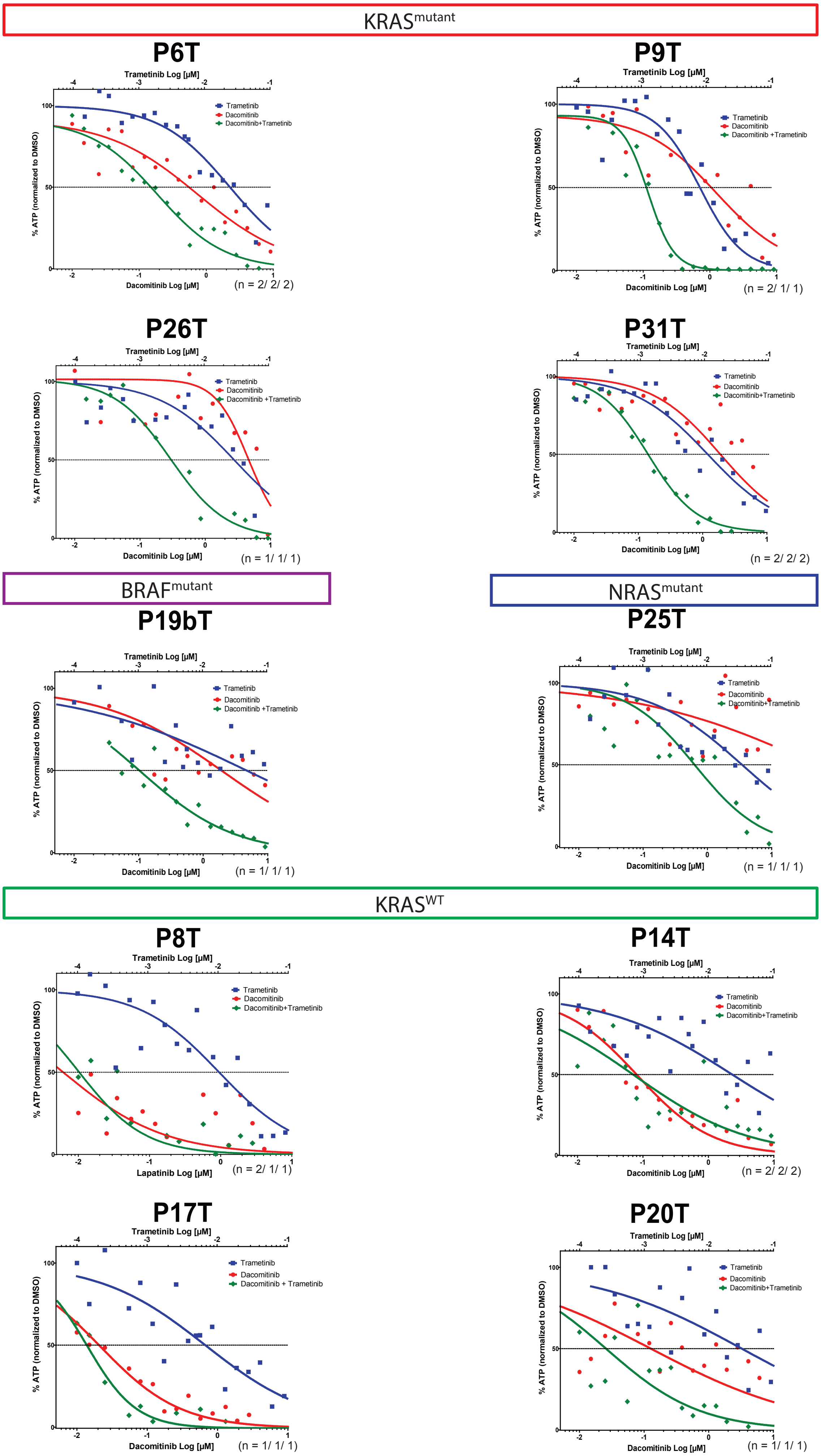

Combination therapy: EGFRi & MEKi

lapatinib & trametinib

KRAS^{mutant}

P6T

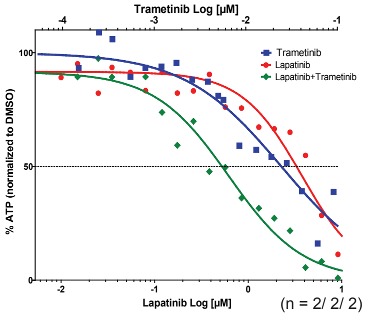

P9T

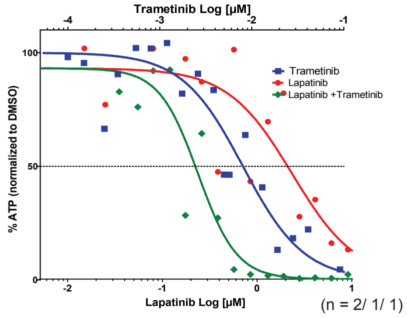

P26T

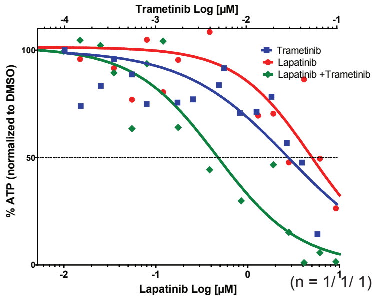

P31T

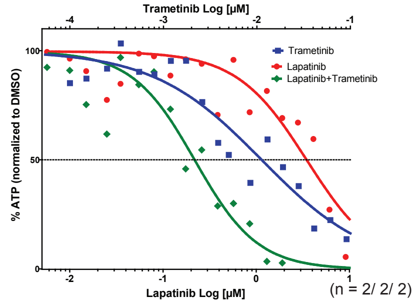

BRAF^{mutant}

P19bT

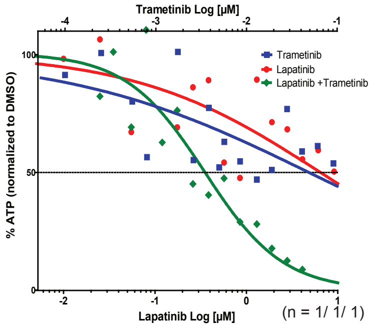

NRAS^{mutant}

P25T

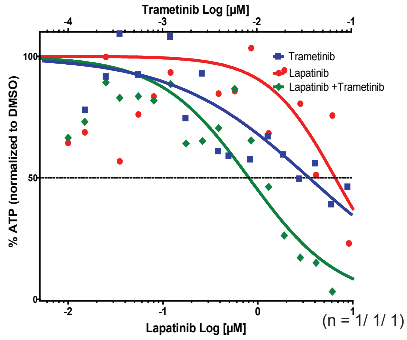

KRAS^{WT}

P8T

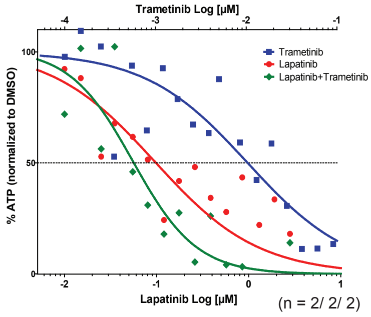

P14T

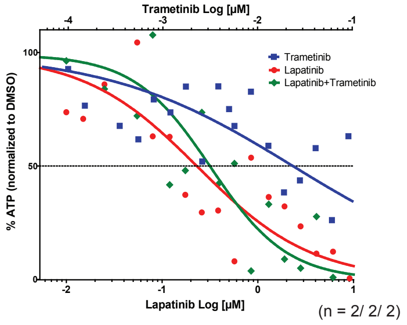

P17T

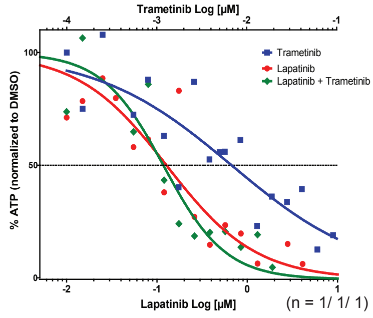

P20T

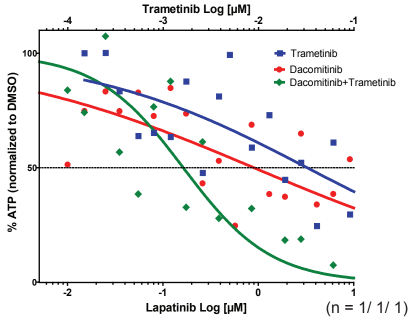

Supplement: Figure 6—source data 1. — A number of biological replicates for each dose-response curve are indicated between parenthesis (first monotherapy/ second monotherapy/ combination therapy). DOI: http://dx.doi.org/10.7554/eLife.18489.025 [file elife-18489-fig6-data1.pdf]
